# Gastrointestinal conditions related to tooth wear

**DOI:** 10.1038/s41415-023-5677-0

**Published:** 2023-03-24

**Authors:** John P. Howard, Laura J. Howard, Joe Geraghty, A. Johanna Leven, Martin Ashley

**Affiliations:** 41415214540001 General Dental Practitioner, Speciality Dentist in Paediatric Dentistry, University Dental Hospital of Manchester, UK; 41415214540002 General Dental Practitioner, Heald Green, Manchester, UK; 41415214540003 Consultant Gastroenterologist, Manchester Royal Infirmary, UK; 41415214540004 Consultant in Restorative Dentistry, University Dental Hospital of Manchester, UK; 41415214540005 Consultant and Honorary Professor in Restorative Dentistry and Oral Health, University Dental Hospital of Manchester, UK

## Abstract

Gastro-oesophageal reflux disease (GORD) is a relatively common condition that occurs in adults and less commonly in children. It develops when the reflux of stomach contents into the oesophagus causes troublesome symptoms and/or complications. Signs and symptoms include heartburn, retrosternal discomfort, epigastric pain and hoarseness, dental erosion, chronic cough, burning mouth syndrome, halitosis and laryngitis. A proportion of patients will, however, have silent reflux. Strongly associated risk factors include family history, age, hiatus hernia, obesity and neurological conditions, such as cerebral palsy. There are different treatment options which may be considered for GORD, consisting of conservative, medical and surgical therapy. Dentists should be aware of the symptoms of GORD and dental signs of intrinsic erosion indicative of possible GORD so that they can question patients about this and, if appropriate, initiate a referral to a general medical practitioner.

## Key points

- Details the common signs and symptoms of gastro-oesophageal reflux disease (GORD) that dentists should be aware of.
- The principle causes of GORD are sphincter incompetence, increased gastric pressure and increased gastric volume. There are a number of medical, diet and lifestyle risk factors which contribute to these causes.
- Management options of GORD consist of conservative, medical and surgical therapy. Dentists should refer patients to their general medical practitioner if undiagnosed GORD is suspected.

## Introduction

Gastro-oesophageal reflux disease (GORD) is a relatively prevalent condition worldwide. A 2020 systematic review stated a prevalence rate of 14.2% in adults throughout Europe^1^^,^^2^^,^^3^ and 14.53% in the UK.^1^ GORD is much less common in children, although it is often seen in neurologically impaired children, such as those with cerebral palsy.^2^

The Royal College of Surgeons' *Clinical guidelines for dental erosion* defines dental erosion as the irreversible softening and subsequent loss of dental hard tissue due to a chemical process of acid dissolution, but not involving bacterial plaque acid, and not directly associated with mechanical or traumatic factors, or with dental caries.^2^

Intrinsic acid as a result of GORD is one of the main causes of erosive tooth wear. It can go undiagnosed in patients who may not realise the symptoms of GORD or the potential significance of this. During dental examinations, signs of intrinsic erosive tooth should alert clinicians to a history of possible GORD.

**Fig. 1:**
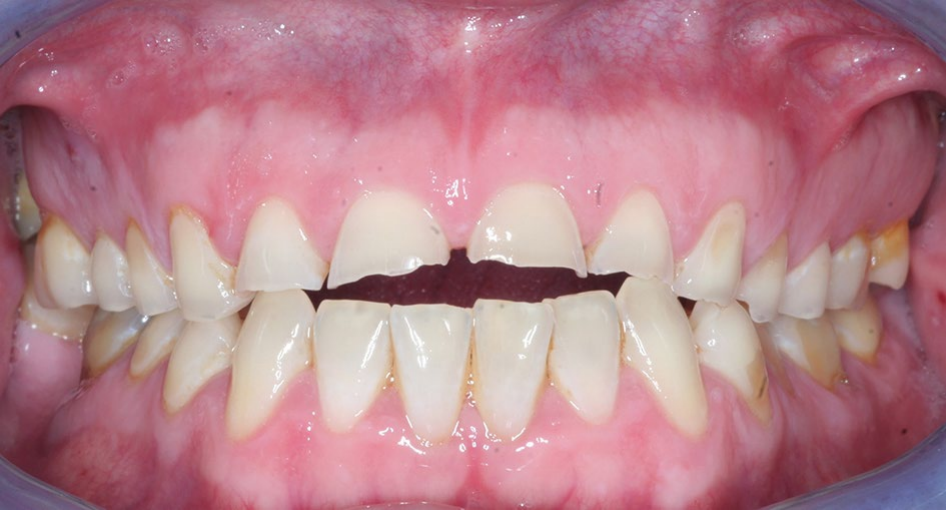
Showing incisal edge wear and buccal wear exposing yellow dentine, with lack of dento-alveolar compensation likely due to the rapid onset

**Fig. 2:**
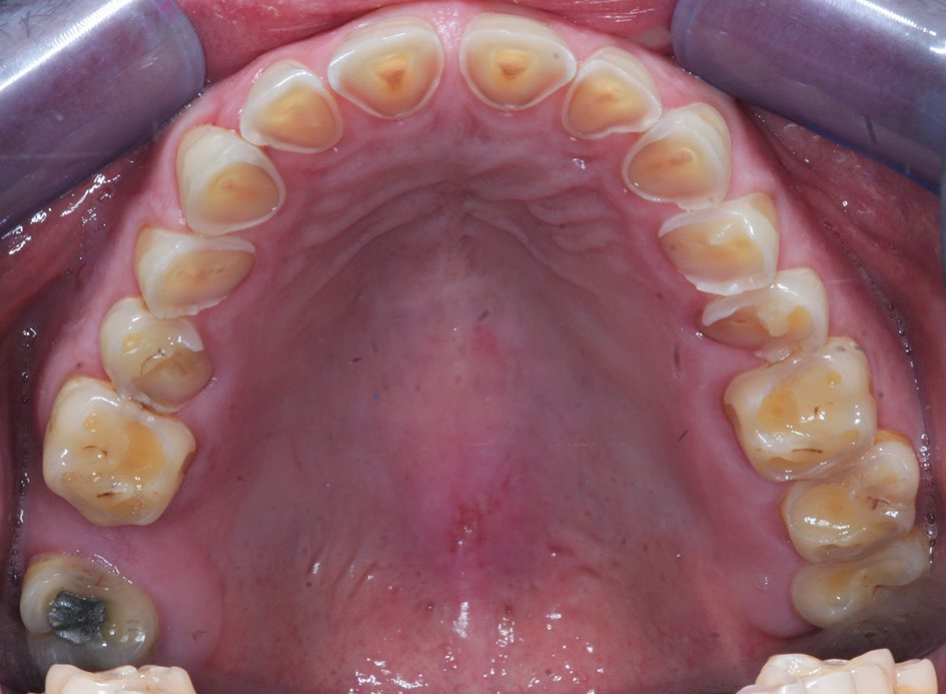
Showing maxillary wear on palatal surfaces, change in occlusal morphology, proud restoration, exposure of dentine with enamel peripheral ring and visualisation of pulp chamber

**Fig. 3:**
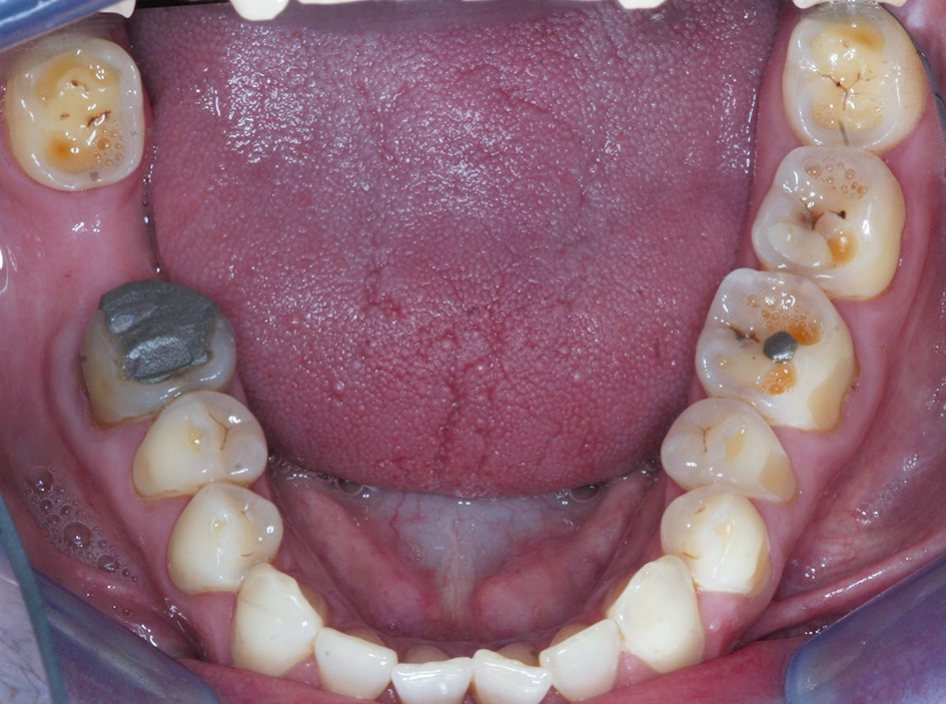
Mandibular wear, showing change in occlusal morphology, hollows and concavities, proud restorations and exposure of dentine

Signs of intrinsic acid-related dental erosion are commonly wear on the palatal aspects of maxillary teeth and the occlusal surfaces of mandibular molars, flattened occlusal contours and cupping of cusp tips. Maxillary buccal cervical erosion may also be present if the patient holds gastric contents in their cheeks. Restorations may stand proud and incisal edges may become grooved. As enamel is eroded away, there may be a blueish tinge, and then teeth may appear darker and yellow dentine starts to shine through, often leaving a peripheral ring of enamel.^2^ Erosive tooth wear tends to leave smoother surfaces compared to other types of wear, although they often occur in combination and a good history will aid diagnosis (Fig. 1, Fig. 2 and Fig. 3 demonstrate these signs, and as such rapid wear had occurred, there has not been any dento-alveolar compensation).

These clinical findings, particularly in the absence of significant dietary acids or a disclosed history of an eating disorder, present an opportunity for dentists to ask more questions about a history of GORD. Patients may not be aware of the symptoms of GORD. Dentists are therefore in a unique role as they may be the first healthcare professional to diagnose possible signs of GORD and question the patient.

## Intrinsic acidic sources

The Montreal definition states GORD is a condition that develops when the reflux of stomach contents into the oesophagus causes troublesome symptoms and/or complications.^4^ As dentists, it is not uncommon to be the first healthcare professional to diagnose a systematic disease through observation of its oral manifestation.^4^^,^^5^

The source of intrinsic acid-related to dental erosion is gastric juice.^2^ Gastric juice is a strongly acidic colourless liquid, with a pH between 1-3. Its immediate secretion is controlled by gastrin release, causing release of the juice that predominately contains hydrochloric acid, the digestive enzyme pepsin and mucus.

There are many risk factors for GORD. These can be separated into strong and weak. Strong risk factors include family history, age, hiatus hernia and obesity. The prevalence of GORD is highest in the 35-59 age group, followed by the over-60s, and then the 18-34-year-olds.^1^ Obese individuals are significantly more at risk of GORD due to the systemic health effects of obesity, as well as the higher intake of food and drinks associated with obesity, such as fatty foods and carbonated drinks.^1^

Weak risk factors include smoking, stress, high alcohol intake, chocolate/spicy foods, caffeinated drinks, asthma and drug induced (for example, nitrates, calcium channel blockers and non-steroidal anti-inflammatory drugs/aspirin). These may increase GORD due to a combination of a slower rate of digestion, irritation of the oesophagus, higher levels of gastric acid secretion, reduction in lower oesophageal sphincter pressure, and/or a delay in gastric emptying.^1^

It is also important to consider vomiting-related eating disorders as a cause for intrinsic acid wear, such as anorexia nervosa, bulimia nervosa, self-induced vomiting and non-specified eating disorders. If this is suspected, thorough history, explanation and then sign-posting to the patient's general medical practitioner is warranted.

## Signs and symptoms of GORD

Systemic symptoms can be either oesophageal, such as heartburn, retrosternal discomfort, epigastric pain and hoarseness,^3^ or extra-oesophageal, such as chronic cough, burning mouth syndrome, halitosis and laryngitis.^1^ Dental symptoms may include pain or sensitivity to hot, cold or sweet substances, and in rare cases, dental abscess due to pulpal exposure. Patients may complain of dental signs, such as yellow discolouration of teeth, poor aesthetics due to volume loss, or the feeling fillings have changed or become proud. It is important to note that nearly 25% of adult patients presenting with extensive palatal erosion had pathological GORD diagnosed but did not have any systemic symptoms of reflux. Therefore, in silent reflux, dental erosion may be the only clinical sign present.^2^^,^^6^

Barrett's oesophagus and oesophageal adenocarcinoma are well-known, rare complications of GORD, and any red flag symptoms, such as dysphagia, odynophagia, globus sensation, anorexia, weight loss and bleeding must be treated seriously.

**Fig. 4:**
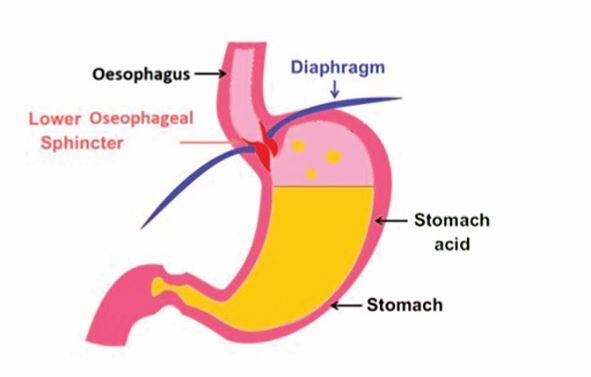
A closed lower oesophageal sphincter, retaining gastric contents *in situ*

**Fig. 5:**
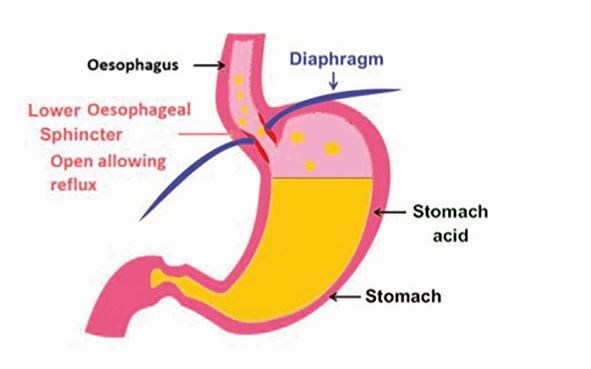
An open lower oesophageal sphincter, allowing retrograde passage of gastric contents

In a normal state, gastric contents are prevented from entering the oesophagus by the anti-reflux mechanism. This consists anatomically and functionally of the lower oesophageal sphincter, extrinsic compression from the diaphragm and the acute angle of His (created between the cardia of the stomach and oesophagus) (Fig. 4). GORD occurs when the gastric contents abnormally exit the lower oesophageal sphincter, allowing passage further up the digestive system, causing damage on their journey (Fig. 5).

A summary of principle causes of GORD can be found in Box 1.^2^

If GORD is suspected, history-taking should include questions about the incidence of belching, heartburn, stomach aches, acidic taste, voice change, hoarseness, chronic cough, vomiting, halitosis, choking and excess salivation to aid onward referral.^7^^,^^8^

Box 1 Principle causes of gastro-oesophageal reflux Sphincter incompetence

- Oesophagitis
- Pregnancy
- Alcohol
- Hiatus hernia
- Diet (spicy/fatty foods)
- Drugs (for example, diazepam)
- Neuromuscular (for example, cerebral palsy)

Increased gastric pressure

- Obesity
- Pregnancy
- Ascites

Increased gastric volume

- After meals
- Obstruction
- Spasm

Reproduced with permission by the Royal College of Surgeons of England^2^

## Management of GORD

For many patients, GORD is a chronic relapsing condition. Dentists should refer (with the patient's permission) to the patient's general medical practitioner, where GORD is suspected as an aetiological factor for presenting with erosive tooth wear.^2^

**Fig. 6:**
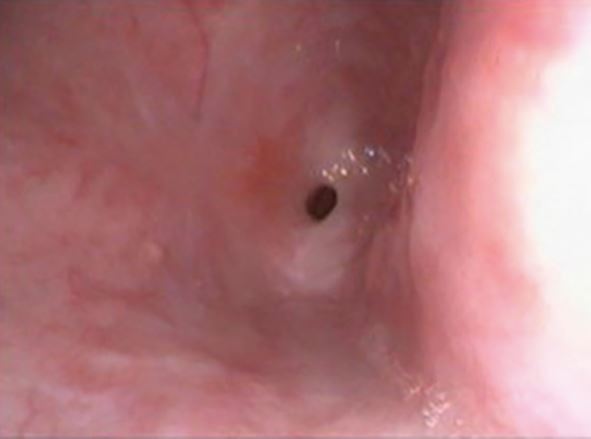
A tight peptic stricture (caused by long-term acid exposure) at the lower oesophageal sphincter region

**Fig. 7:**
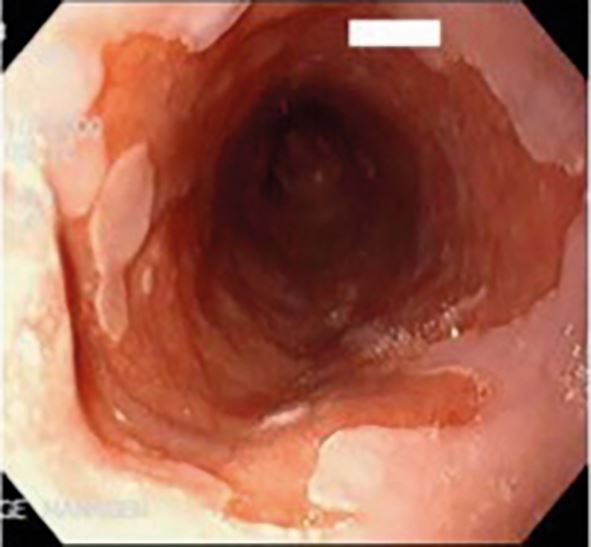
Barrett's oesophagus of the lower oesophagus, a premalignant condition caused by long-term acid exposure

**Fig. 8:**
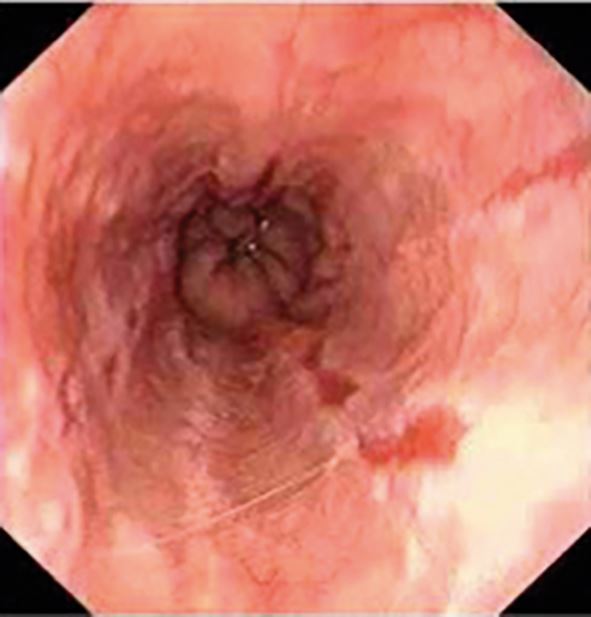
Grade A oesophagitis caused by excess acid exposure within the lower oesophagus

Diagnosis is often clinical; however, upper endoscopy is warranted for red flag symptoms, or no improvement after eight weeks of medical treatment. The role of endoscopy is to confirm diagnosis (erosion/ulcerations or non-erosive reflux disease), exclude atypical causes (eosinophilic esophagitis, candida, herpes simplex) and diagnose complications (Barrett's oesophagus, stricture, adenocarcinoma) (Fig. 6, Fig. 7, Fig. 8).

When initial investigations are inconclusive or symptoms persist despite standard management, further investigations may be required in tertiary care. These include assessing the pH in the lower oesophagus (to look at absolute amounts of reflux and concordance with symptoms), high-resolution manometry of the oesophagus (to exclude motility disorders such as achalasia, which can mimic reflux), and also assess whether motility is sufficiently preserved to allow more invasive treatments, such as anti-reflux surgery (for example, a Nissen's fundoplication) or newer treatments (such as magnetic augmentation of the lower oesophageal sphincter).

Management options for GORD consists of conservative, medical and surgical therapy.

Conservative methods include life-style modification, such as dietary changes, smoking cessation and weight loss management. Dentists are in a good position to give specific dietary advice for GORD-related tooth wear, such as reduced frequency of dietary acid intake (especially at bedtime), avoidance of reflux-provoking foods, and the use of sugar free chewing gum after an acid exposure to increase salivary flow and encourage tooth remineralisation.^2^^,^^9^

Medical methods are most commonly antisecretory drugs, such as protein pump inhibitors (for example, omeprazole) although H2 blockers (for example, ranitidine) and newer therapies, such as K+ competitive acid blockers (for example, vonoprazan), are also used.

Antacids to neutralise acid, and alginates which precipitate into a gel on contact with acid, are often used as over-the-counter self-medication by patients. It is important to note that although most antacids and alginates are sugar-free, not all of them are. As alginates are commonly taken last thing at night (to neutralise acid movement when the patient is prone), this may add a cariogenic risk factor, which patients should be informed of. Dentists can provide interventions to help with remineralisation and sensitivity of teeth, such as the use of high fluoride toothpastes and mouthwashes, GC Tooth Mousse, regular application of fluoride varnish and application of resin sealants or dentine bonding agents to provide temporary sensitivity relief.^2^^,^^9^

Lastly, if other therapies are unsuccessful, surgery may be offered. There are a number of options for this, such as fundoplication (either surgical or endoscopic), and other endoscopic procedures. Each of these have general indications and side-effects that a patient will discuss with their surgical team.

## Conclusion

Dentists may be the first clinicians to detect signs of GORD and therefore play an important role in screening for GORD. Patients may have silent reflux, not be aware of the symptoms of GORD, or that the frequency with which they are experiencing GORD might be a cause for concern. Dentists therefore can have a positive role in providing patient education and offering referral to the general medical practitioners. Early detection may prevent long-term gastro-oesophageal complications and progression of the tooth wear condition.
